# Provision of High Protein Foods Slows the Age-Related Decline in Nutritional Status in Aged Care Residents: A Cluster-Randomised Controlled Trial

**DOI:** 10.1007/s12603-022-1868-7

**Published:** 2023-01-10

**Authors:** Sandra Iuliano, S. Poon, J. Robbins, X. Wang, M. Bui, E. Seeman

**Affiliations:** 1 Department of Endocrinology, University of Melbourne / Austin Health, Heidelberg Repatriation Hospital, Waterdale Road, West Heidelberg, 3081, Melbourne, Victoria, Australia; 2 School of Population and Global Health, University of Melbourne, Melbourne, Australia

**Keywords:** Aged care, high-protein foods (milk, cheese, yoghurt), malnutrition

## Abstract

**Objectives:**

Malnutrition, particularly protein insufficiency, is common in institutionalised older adults and increases morbidity, mortality, and costs. We aimed to determine whether 12 months supplementation using high-protein foods (milk, cheese, yoghurt) prevents malnutrition in older adults.

**Design:**

Cluster randomised control study.

**Setting:**

Sixty Australian aged care facilities.

**Participants:**

Older adults living in aged care homes (n=654, mean age 86.7±7.2 years, 72% females).

**Intervention:**

Facilities randomly allocated to a high-protein (n=30 intervention) or regular (n=30 controls) menu.

**Measurements:**

Nutritional status assessed using the Mini Nutrition Assessment (MNA) tool and fasting morning blood samples (n=302) assayed for haemoglobin (Hb) and albumin. Food intake was monitored 3-monthly using visual plate waste assessment. Measurements at baseline and month 12 were analysed using random effects model accounting for clustering (facility), repeated measure and confounders.

**Results:**

Addition of 11g of protein as 1.5 servings of high-protein foods daily preserved nutritional status that deteriorated in controls [MNA screen (−0.68, 95%CI: −1.03, −0.32, p<0.001) and total (−0.90, 95%CI: −1.45, −0.35, p=0.001) scores], resulting in group differences in MNA screen (0.62, 95%CI: 0.17, 1.06, p=0.007) and total (0.81, 95%CI: 0.11, 1.51, p=0.023) scores and group difference in Hb (3.60g/L, 95%CI: 0.18, 7.03, p=0.039), the net result of preservation with intervention (0.19g/L, 95%CI: −2.04, 2.42, p=0.896) and a decline in controls (−3.41g/L, 95%CI: −6.01, −0.82, p=0.010). No group differences were observed for serum albumin.

**Conclusion:**

Consumption of high-protein foods is a pragmatic approach to maintaining nutritional status in older adults in aged-care.

## Introduction

**A**s the population ages, by 2057, 25% of the population will be older than 65 years, increasing the demand for aged care (1). Malnutrition is common in older adults in aged care with ~72% of residents at risk of malnutrition (2, 3). Insufficient protein intake contributes to malnutrition, increasing morbidity, mortality, and health care costs due to increased risk of frailty and falls, pressure sores, impaired wound healing, delayed recovery from illness, and protracted hospital stay (4, 5, 6, 7, 8).

Institutionalised older adults often receive only 25–50% of recommended levels of high-protein foods i.e., 1 compared to 4 serving of dairy (milk, yoghurt, cheese) and 1 compared to 2 servings of ‘meat' (lean meat, poultry, eggs, seafood, legumes, nuts and seeds), resulting in inadequate protein intakes (< 0.8 grams per kg body weight) (9). When nursing home residents are provided with recommended amounts of protein-rich foods, protein intake averaged 82.6g/day, achieving 130% of recommended levels (10). Supplementing aged care residents with 2 additional servings of high-protein foods daily improved protein intake by 25±5 g, achieving a daily intake of 74±16g protein (137±39% of protein requirements), compared with non-supplemented residents in whom daily protein intake remains at 56±15g, and below protein requirement (11).

Given milk, cheese and yoghurt are high-protein foods that are familiar to older adults, palatable, inexpensive, and widely available, we aimed to determine whether increasing provision of these foods to the recommended four servings daily will reduce malnutrition risk in older adults in aged care. The primary objective was a reduction in malnutrition risk based on the Mini Nutrition Assessment (MNA) tool and the secondary objective was improvements in serum measures of nutritional status.

## Materials and Methods

This was part of a 2-year cluster-randomised controlled trial involving 60 aged care facilities from metropolitan Melbourne and regional Victoria, Australia, with primary and secondary outcomes published recently (12). In brief, facilities recruited between December 2013 and August 2016 were placed in blocks based on organisation and stratified by location then randomised in a 1:1 ratio to intervention (n = 30 facilities; high-protein menu) or control (n = 30 facilities; regular menu). Organisations had between 2 to 10 facilities and randomisation was done within an organisation. Randomisation was computer generated and performed by an independent statistician who provided the concealed group allocation to the principal investigator who informed facility management of their group allocation.

**Figure 1:**
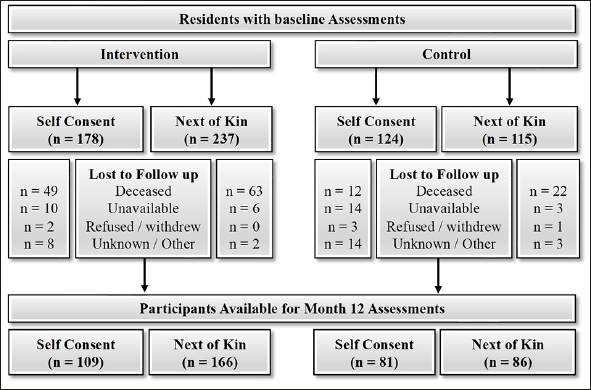
Flow of older adults in aged care participating in the first 12 months of a 2-year cluster-randomised controlled trial of consumption from a high-protein (intervention) or regular (control) menu

Inclusion criteria for facilities were (i) accreditation by the Australian Aged Care Quality Agency to ensure similar standards of care, and (ii) they accommodated ambulant residents. Facilities recruited varied in size; small (up to 50 beds), medium (51–100 beds) and large (>100 beds) and types of providers (for-profit, charitable, privately owned), providing a good representation of aged care homes. The sex distribution and age of residents were indicative of the national average (13). Residents were informed of the facility's involvement in the study during resident meetings, from which 302 residents self-consented and 354 were consented by a next of kin to have dietary intake and nutritional status assessed and medical records reviewed (Figure 1). Serum sampling was restricted to the 300 residents providing written consent. Once care homes were assigned to their group only the principal investigator, research dieticians, facility management and food service staff were aware of group allocation. Research staff involved with testing and data acquisition were blinded to group allocation. Residents were not informed of the facility's group allocation.

All aged care facilities were equipped with commercial kitchens that operated a cook-fresh food service, with food prepared on-site based on 4-week (monthly) menu cycles. A typical daily menu consisted of a continental-style (and very occasionally hot) breakfast, a midday meal of a usually hot meat-based dish and dessert, an evening meal of soup and choice of a hot or cold dish and dessert, with snacks provided during the morning, afternoon, and evening. Control sites continued with their usual food service. Intervention sites were assigned qualified dieticians specialised in food service who worked with food service staff to assist with menu modifications to incorporate additional servings of milk, cheese and/or yoghurt (all types and all fat contents) into daily menus (9). Consumption of the high-protein foods at recommended levels provide protein within approved guidelines and suggested levels (1.0 – 1.5 g per kg BW) (14, 15). As part of routine care residents with special dietary needs (e.g., those with renal failure) are prescribed special diets so would not be affected by the intervention.

Based on the Australian Guide to Healthy Eating servings are defined as; milk (250 ml), cheese (40 g) and yoghurt (200 g) (9). Butter, cream, and ice-cream contain limited protein, so not provided as part of the intervention. Changes to menus included (i) substitution of snacks with dairy-based alternatives, (ii) addition of milk, cheese, or yogurt to meals, (iii) fortification of foods with milk powder and (iv) recipe modifications to increase the protein content using the foods described. Residents with lactose intolerance were provided with lactose-free milk. All additional high-protein foods were provided to intervention facilities via a single commercial food distribution company (BidFoods, Australia) and all invoice data recorded to corroborate adherence to intervention foods. Intervention foods was provided to all residents at intervention sites. Control facilities continued providing food and beverages from regular menus with no changes as determined by dietary intake and invoice data.

Dietary intake and adherence to intervention was assessed by trained research dieticians over two random days using the validated method of visual estimation of plate waste (16). A digital food scale (accuracy ±1g, Soehnle Page Profi) was used to weigh standard serving of all components of meals and snacks. A seven-point scale was used to represent portions of each food consumed (or remaining); 0=no food remaining, ±M = 1 mouthful remaining, ¼ = 25% remaining, ½ = 50% remaining, ¾ = 75% remaining, −M = 1 mouthful consumed (90% remaining), 1 = no food eaten. Meals served were rated against the standard meal (medium = 100%); small serving = 75%, large serving = 125%, extra-large serving = 150%. Consumption was calculated as the difference between the amount served and wasted. Dietary information, specifically protein, energy and servings of milk, yoghurt and / or cheese, was collected at baseline and every 3 months for 12 months. The Schofield equation was used to estimate energy requirements using estimated height from ulna length and applying a physical activity level of 1.20 (17). All dietary analyses were performed using the computerised Foodworks dietary analysis program (2009, Professional XYRIS Software, Queensland, Australia).

A qualified dietician on the research team assessed nutritional status in all consented residents using the Mini Nutritional Assessment (MNA) tool (Nestlé Nutrition Institute, Vevey, Switzerland), a validated tool comprising 18 questions related to nutritional status, with a maximum score of 30 (18). Malnutrition was defined as a score of < 17; at risk of malnutrition for scores between 17 to 23.5; and normal nutritional status for scores from 24 to 30. The first 6 questions of the MNA tool constitute the MNA screening component (short form) that has a maximum score of 14. Malnutrition using the short form was defined as a score between 0 to 7; at risk of malnutrition for scores between 8–11; and normal nutritional status for scores from 12 to 14. Both the short and long form of the MNA tool have been validated (19). Staff assisted with responses, and objective measures (e.g., weight changes) were obtained from medical records maintained at the care homes. This enabled accurate assessment in all residents including those with cognitive impairment. Assessments were performed by the same dietician at baseline and month 12.

Fasting morning blood samples were taken on-site by a qualified phlebotomist (Melbourne Pathology, Australia) at both control and intervention facilities at baseline and month 12. Samples were assayed for haemoglobin (Roche Sysmex XN20 analyser) and albumin (Roche Sysmex and Cobas 701; Roche Diagnostics, Indianapolis, IN); coefficient of variation: 1%–5%, both validated markers of nutritional status, with albumin more effective in clinically stable older adults (20, 21). Normal reference ranges were: haemoglobin (females 120–165g/L, males 130–185g/L) and albumin (>80 years; females and males, 32–43g/L; 50–79 years; females 33–44g/L; males 34–45 g/L).

Nutritional outcomes (MNA score and serum measures) were tertiary outcomes for the overall study (12). Based on the distribution of MNA scores in aged care residents, 141 participants per group were required to detect a 1 ± 3 point difference between groups in MNA score with 80% power at the p < 0.05 level (3). Data were analysed using Stata software, version 14.2 (Stata Corporation Inc., College Station, TX, USA). Effect of intervention on each mini nutrition assessment (screen score and total score) and biochemistry (haemoglobin and albumin) was assessed comparing change from baseline to follow-up (12 months) in the intervention group minus the change from baseline to follow-up in the control group (22). A model involves additive effect of indicator variable group (0 = control; 1 = intervention), time (0 = baseline, 1 = follow-up) and interaction between these two variables, where the estimated coefficient term for interaction is known as difference-in differences estimate. Estimation was carried out using random effects model, accounting for correlation within facility and repeated measures. P-value (p) < 0.05 were considered statistically significant. The study was approved by the Austin Hospital Human Research Ethics committee (project number 04958) and is registered on the Australian New Zealand Clinical Trials Registry (ACTRN12613000228785).

## Results

Baseline characteristics were similar between groups (Table 1). Residents on average had 10 medical conditions and were prescribed 12 medications. The majority had a diagnosis of cardio-vascular disease and just under half had some form of cognitive impairment. By 12 months, 442 residents remained after loss of follow up due to death (n = 160), residents being unavailable (n = 18), refused testing or withdrawal from study (n = 6) or other / unknown reasons (n = 28) (Figure 1).

**Table 1:** Baseline characteristics of aged care residents at intervention (high-protein menu) and control (regular menu) care homes. Data expressed as mean ± standard deviation unless otherwise stated

	**Intervention (n = 415)**	**Control (n = 239)**
Age (years)	86.7 ± 7.5	86.0 ± 6.8
Sex, n (%): Male	120 (28.9)	65 (27.2)
Female	295 (71.1)	174 (72.8)
Height (m)^1^	1.60 ± 0.08	1.61 ± 0.08
Weight (kg)	66.9 ± 15.2	68.3 ± 15.9
BMI (kg/m^2^)	25.7 ± 5.2	26.2 ± 5.5
Medications*	11 (8–14)	12 (9–16)
Medical Conditions*	10 (7–13)	10 (8–14)
CVD (%)	59.6	62.6
Cognitive Impairment (%)	48.8	44.5
Nutritional status		
MNA Score: Screen^2^	9.7 ± 2.3	10.2 ± 2.2
Total^3^	20.3 ± 3.9	21.2 ± 3.5
MNA Categories; n (%)		
Malnourished	71 (17%)	26 (11%)
At Risk of Malnutrition	273 (66%)	158 (66%)
Normal nutrition status	71 (17%)	55 (23%)
25(OH)D (n/mol/l) **	72.2 ± 28.5	73.6 ± 25.5
Albumin (g/l)^4^ **	35.8 ± 3.7	36.3 ± 3.8
Hb (g/l)^4^ **	127.7 ± 14.5	127.4 ± 17.3
Daily Dietary Intake		
Protein (g) ^A^	58 ± 16	56 ± 16
Energy (kJ) a	6815 ±1703	6631±1687
Dairy foods (servings) a#	2.0 ± 1.0	1.7 ± 1.0

BMI = Body Mass Index; CVD = Cardiovascular Disease; MNA = Mini Nutrition Assessment; 25(OH)D = 25-hydroxy vitamin D; Hb = haemoglobin; 1. Determined from ulna length; 2. MNA Screening Score maximum = 14; 3. MNA Total Score maximum = 30; 4. Reference Ranges: Albumin 32–45g/L; Haemoglobin Male 130–180g/L, Female 120–160g/L; *median (interquartile range); ** n 178 intervention and 124 control; a n 391 intervention and 226 control; # 1 Dairy Standard Serve (500–600kJ): 1 cup (250mL) fresh, UHT long life, reconstituted powdered milk or V cup (120mL) evaporated milk or 2 slices (40g) cheese or V cup (120g) ricotta cheese or M cup (200g) yoghurt (8)

**Figure 2:**
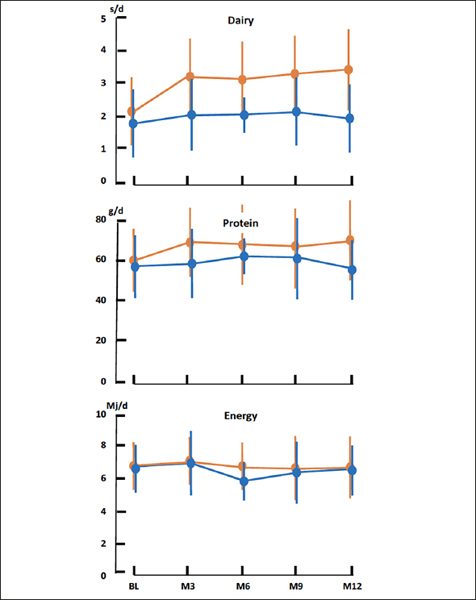
Adherence to dietary intervention, and dietary intake data assessed quarterly in older adults in aged care consuming from a high-protein (intervention; orange line) or regular (control; blue line) menu

Adherence to the dietary intervention and regular food intake were measured quarterly (Figure 2). Intervention increased servings of milk, yoghurt and / or cheese from 2.0 ± 1.0 at baseline to 3.5 ± 1.5 servings/day at month 12 (p < 0.001) that remained unchanged in controls (1.7 ± 1.0 servings/ day at baseline vs. 1.9 ± 1.0 servings/day at month 12, p = 0.232). At month 12 protein intake was 11g (95% CI 8, 14, p < 0.001) higher with intervention and remained unchanged in controls 1 g, (95%CI: −2, 4, p = 0.413). Energy intake remained unchanged in both groups. No adverse gastrointestinal events were observed with intervention.

As shown in Table 2, age-adjusted between group differences were observed for MNA screen (0.62, p = 0.007), and total (0.81, p = 0.023) scores, the result of no change with intervention and decreases in controls (screen score; −0.68, p < 0.001 and total score; −0.90, p = 0.001). During the intervention period, a greater proportion of controls deteriorated in nutritional status based on MNA categories (well nourished, at risk of malnutrition, malnourished) than those in the intervention group (32% versus 22%, p = 0.019).

**Table 2:** Difference-in-differences estimate of intervention effects of consumption from a high-protein versus regular menu by aged care residents using random effects model accounting for clustering (facility) and repeated measures

	**Baseline**				**Follow-up**				**Difference**			
	**N**	**Coef**	**se**	**p-value**	**N**	**Coef**	**se**	**p-value**	**Coef**	**se**	**p-value**	**95% CI**
Screen Score*												
Control	239	12.0	0.92		167	11.3	0.92		−0.68	0.18	<0.001	(−1.03, −0.32)
Intervention	415	11.8	0.91		275	11.7	0.91		−0.06	0.14	0.672	(−0.33, 0.21)
Difference		−0.19	0.26	0.454		0.42	0.28	0.133	0.62	0.23	0.007	(0.17, 1.06)
Total Score*												
Control	235	24.5	1.63		164	23.6	1.63		−0.90	0.28	0.001	(−1.45, −0.35)
Intervention	412	24.3	1.62		268	24.2	1.62		−0.09	0.22	0.665	(−0.52, 0.33)
Difference		−0.26	0.44	0.560		0.55	0.48	0.247	0.81	0.36	0.023	(0.11, 1.51)
Haemoglobin (g/L)**												
Control	123	135.5	1.91		81	132.1	2.05		−3.41	1.32	0.010	(−6,01, −0.82)
Intervention	177	133.9	1.70		108	134.1	1.82		0.19	1.14	0.869	(−2.04, 2.42)
Difference		−1.65	1.81	0.361		1.95	2.07	0.346	3.60	1.75	0.039	(0.18, 7.03)
Albumin (g/L)**												
Control	124	37.2	0.46		81	35.8	0.50		−1.40	0.42	0.001	(−2.22, −0.57)
Intervention	178	36.9	0.42		109	35.7	0.46		−1.18	0.36	0.001	(−1.89, −0.48)
Difference		−0.38	0.47	0.422		−0.16	0.56	0.768	0.22	0.55	0.697	(−0.87, 1.30)

Adjusted for *age or **sex; Coef = estimate coefficient from fitting random effects model with interaction between group (control versus intervention) and time (baseline and follow-up) to the data; se = standard error; CI = confidence interval; difference-in-differences estimates and their standard error and p-value for each variable are in bold.

Sex-adjusted group difference in favour of intervention was observed for haemoglobin (Hb) (3.60 g/L, p = 0.039). For residents consuming the high-protein menu, Hb remained unchanged and decreased in controls (−3.41g/L, p = 0.010). Serum albumin levels declined in both the intervention (−1.18g/ L, p = 0.001) and control (−1.40g/L, p = 0.001) groups with no group differences observed (0.22 g/L, p = 0.697).

## Discussion

Increasing consumption of high-protein foods by 1.5 servings daily, providing ~10 g extra protein daily, maintained nutritional status and serum haemoglobin levels that declined in controls. No group differences were observed for serum albumin levels.

The increased consumption of the high-protein foods maintained nutritional status without increasing energy intake. One strategy used was high-energy, nutrient-poor ‘discretionary' foods (e.g., cakes and sweet biscuits) were replaced by high-protein alternatives. High-energy foods may achieve weight gain but have limited nutritional quality. For example, Leslie et al. (2013) supplemented undernourished aged care residents by adding double cream and butter to meals that resulted in increases in body weight and fat intake but not protein intake as these foods provide little protein (23).

The consumption of an additional 1.5 servings of highprotein foods daily maintained but did not improve Mini Nutrition Assessment score. We previously observed that among 215 older aged care residents (mean age 85.8 years) with inadequate intakes of high-protein foods, modelling suggested that each serving of milk, yoghurt or cheese was associated with a 1 point increase in Mini Nutrition Assessment score (3). Therefore, if residents consumed the recommended 4 servings daily of these foods, they would, on average, achieve normal nutritional status (MNA score >24 points) (3, 9). However, within the Mini Nutrition Assessment tool only 11 of the 18 questions may be directly (or indirectly) influenced by improved protein intake while the remaining questions such as number of medications, place of residency and feeding assistance are not amenable to improvement in protein intake.

Provision of additional high-protein foods prevented the decline in haemoglobin levels observed in controls. Haemoglobin is considered a usual biomarker of malnutrition with lower haemoglobin levels observed in malnourished people compared to those with normal nutritional status (20). In contrast, despite mean serum albumin levels being on the lower end of the normal range (34–54 g/L) they were unaltered with intervention. Similarly, Van Wymelbeke et al. (2016) observed no change in serum albumin levels when malnourished residents consumed brioche enriched with protein, despite improvements in energy and protein intakes (24). Therefore, in this group of older adults in care homes, serum albumin level may not have been a suitable indicator of nutritional status as it is affected by non-nutritional factors such as inflammation, infection and chronic liver failure (25).

Compliance with this food-based intervention was sustained for 12 months and we reported this compliance continued for a further 12 months (12). In contrast, compliance with oral protein supplementation declines after 6 months (26). Moreover, during a 10-week intervention in frail nursing home resident, habitual intake declined in those randomised to consume oral nutritional supplements, resulting in no change to total energy intake (27). Improving food quality by providing adequate protein is a pragmatic approach to maintaining nutritional adequacy in older adults in residential aged care.

This practical approach of incorporating high-protein foods into menus required minimal time and skills to prepare. The foods were familiar to resident and were palatable, features that improves voluntary intake in this population (28). Moreover, the high-protein foods used contributed other nutrients such as calcium, vitamins B2 & B12, zinc and leucine, a potent stimulant for muscle protein synthesis (28, 29).

Preventing malnutrition is likely cost saving. For example, the cost of malnutrition in aged care homes is estimated at €11,800 per resident per year for those defined as malnourished and €7,800 for those at risk of malnutrition (30). Moreover, malnourished residents are 1.7 times more likely to fall than well-nourished residents (6). Falls are estimated to cost from €193 for a non-injurious fall to €10,170 for a highly injurious fall, and we reported an 11% reduction in falls with this intervention (6, 12).

Limitations of the study include the high attrition rates due to advanced age and number of co-morbidities. Acute illness, hospitalisation and presence of comorbidities that may contribute to malnutrition risk were monitored but be controlled for. The MNA tool may miss subtle changes in nutritional status such as slow but progressive weight loss, and some questions such as number of prescription medications and place of residency are not amenable to change using a food-based intervention. The efficacy of other high-protein foods such as lean meat, poultry, seafood, eggs, legumes, nuts, seeds, and plant-based alternatives was not evaluated. Furthermore, a cost-benefit analysis of this food-based approach was not performed.

In conclusion, consumption of high-protein foods is a pragmatic dietary approach to maintaining nutritional status in older adults in residential aged care and should be incorporated into food-based policies and guidelines for aged care homes.
